# The effects of prediabetes and type 2 diabetes mellitus on cognitive status, physical function, and fatigue in older adults: a case-control study

**DOI:** 10.1007/s11845-026-04483-z

**Published:** 2026-06-13

**Authors:** Kevser Gürsan, Emre Cengi̇z, Elif Gür Kabul, Atilla Okan Kılıç, Merve Uçar Toros, Bora Toros

**Affiliations:** 1 Faculty of Health Sciences, Physiotherapy and Rehabilitation Department, Usak University, Uşak, Türkiye; 2 Department of Endocrinology and Metabolism, Usak Training and Research Hospital, Usak, Türkiye

**Keywords:** Type 2 Diabetes, Prediabetes, Older Adults, Cognition, Fatigue, Physical Function

## Abstract

**Objective:**

The purpose of this study was to compare the cognitive status, physical function, and fatigue levels of Type-2 Diabetes Mellitus (T2DM), prediabetes, and healthy older adults.

**Methods:**

Volunteers aged 60 and over were included in the study. Participants divided into four groups according to hemoglobin A1c (HbA1c) levels: healthy, prediabetes, controlled T2DM, and uncontrolled T2DM. Cognitive function, spatial ability, functional mobility with and without dual-task conditions, reaction time, fatigue, and biochemical parameters were evaluated.

**Results:**

A total of 94 participants (53 female, 41 male, mean age:67.12±5.84 years) were included. Significant group differences were observed in attention and delayed recall subdomains and total Montreal Cognitive Assesment scores (p<0.05). Additionally, significant differences were found in functional mobility, dual-task performance, foot reaction time, and fatigue levels (all p<0.05). Older adults with uncontrolled T2DM showed poorer cognitive and motor performance than healthy older adults. Adjusted analyses demonstrated that cognitive performance, reaction time, and fatigue-related differences remained significant after controlling for potential confounders, whereas functional mobility-related outcomes were attenuated. Among the biochemical markers, fasting blood glucose levels showed a significant difference among the groups (*p*<0.001).

**Conclusions:**

Uncontrolled T2DM in older adults is associated with impairments in attention, memory, mobility, and motor performance, while fatigue was increased across prediabetic and diabetic individuals. These findings highlight the multidimensional functional impact of dysglycemia in older adults.

## Highlights

This study aims to clarify how the dysglycemia spectrum (prediabetes, controlled T2DM, uncontrolled T2DM) relates to cognition, mobility, and fatigue in community-dwelling older adults.

The study question was whether cognitive subdomains, dual-task mobility, foot reaction time, and fatigue differ across HbA1c-defined groups beyond demographic and comorbidity factors.

Results revealed that uncontrolled T2DM had lower MoCA attention, delayed recall, and total scores; slower TUG performance and prolonged foot reaction times; and fatigue was elevated in both prediabetes and T2DM.

These findings highlight potential associations between dysglycemia and impairments in cognition, mobility, reaction time, and fatigue, and suggest areas that may warrant further investigation in future longitudinal and interventional studies.

## Introduction

In recent years, the increasing number of older adults worldwide has led to an increase in the number of chronic metabolic disorders. Type 2 diabetes mellitus (T2DM) is the most common disorder in the older adults [1]. T2DM is a chronic metabolic disease characterized by persistent hyperglycemia, resulting from impaired insulin secretion and/or insulin resistance [2]. Prediabetes, defined as the transitional phase of this metabolic disease, is characterized by glucose levels above normal and is a significant risk factor for T2DM [3].

The symptoms of T2DM can negatively affect many different body systems [4]. T2DM negatively affects not only cognitive functions but also physical functioning [5, 6]. T2DM-induced imbalances such as impaired insulin levels in the cerebral circulation, increased glycemia, neuroinflammation, oxidative stress levels, and macro- and microvascular complications can impair cognitive functions [6, 7]. Indeed, along with the decline in cognitive functions inherent in aging, T2DM, accompanied by this condition, can lead to significant deterioration in older adults’ memory, reasoning, language, sensory processing, attention, comprehension, analysis, interpretation, learning, motor skills, and spatial abilities. This can limit the activities of older adults and threaten their independence [3, 7]. However, not only T2DM but also prediabetes can affect individuals’ cognitive functions [5].

It is noteworthy that T2DM not only negatively impacts cognitive functions but also negatively impacts physical functionality [8]. Complications such as decreased muscle strength and volume, decreased walking speed, and balance and physical performance losses can be frequently observed in older adults with T2DM [4, 9]. These complications may limit activities of daily living and reduce quality of life in older adults [10]. In addition to these limitations, fatigue symptoms are also common in older adults.

Fatigue leads to decreased performance and decreased motivation to exercise [11]. Although fatigue can result from many causes, it is associated with the impaired glycemic balance of T2DM [12]. Studies have addressed the cognitive status, physical functionality, and fatigue levels of older adults with T2DM separately, but their interrelationships have not been examined [13]. Furthermore, case-control studies identifying and examining the relationships between advanced stages of T2DM, prediabetes, and healthy older adults in different areas are limited [7, 9]. Therefore, this study aimed to holistically evaluate the physical, cognitive, and fatigue levels of patients with T2DM at different stages and healthy older adults using subjective and objective scales, performance, and laboratory tests. The results of this evaluation may contribute to a better understanding of the functional consequences of dysglycemia in older adults and may help guide future preventive and interventional research.

## Materials and methods

### Study design

This study was designed as a comparative case-control study in line with the planned objective. Four groups were compared: Non-Diabetic, Prediabetes, Controlled Diabetes, and Uncontrolled Diabetes. The study was reported according to the Strengthening the Reporting of Observational Studies in Epidemiology guideline [14].

### Study setting and time

The study was conducted with older adults presenting to the Endocrinology Outpatient Clinic of Uşak Training and Research Hospital between April 15, 2025, and August 15, 2025. All participant assessments were conducted face-to-face. Laboratory tests were also performed at the same hospital’s accredited biochemistry laboratory.

### Study permits

The study was approved by the Non-Interventional Clinical Ethics Committee of XXX University (Date: 06/02/2025, Decision number:11). Study permits were also obtained from the XXXX Training and Research Hospital, where the study was conducted (Date: 31/01/2025, Decision Number: E-45786011-799−267059530). The study was conducted in accordance with the Declaration of Helsinki. Informed consent was obtained from each study participant. The study was registered on the clinicaltrials.gov.tr system under the number NCT06953609.

### Participants

Participants were volunteers aged 60 and over who presented to the Endocrinology Unit of a Training and Research Hospital. Participants were divided into four groups based on their hemoglobin A1c (glycosylated hemoglobin, HbA1c) levels: healthy (HbA1c: 4–5.6%), prediabetes (HbA1c: 5.7–6.4%), controlled T2DM (HbA1c: 6.5–7.5%), and uncontrolled T2DM (HbA1c > 7.5%). Participants were included as follows: age 60 or older, appropriate HbA1c levels, volunteer status, and informed consent. Exclusion criteria: Neurological (Alzheimer’s, Parkinson’s) and psychiatric (major depression) diseases, hearing and vision loss, orthopedic gait problems, a history of advanced cognitive impairment/dementia, uncontrolled thyroid disorders, B12/folic acid deficiency, anemia, hospitalization or infection within the last 3 months, malignancy, lung disease, nephropathy, retinopathy, alcohol or substance abuse, and psychotropic medication use (antidepressant, anticonvulsant, etc.).

### Sampling method and group definitions

A purposive sampling method was used to recruit participants to the study group. Older adults who consecutively presented to the endocrinology outpatient clinic and met the inclusion criteria were included in the study. Participants were classified according to their HbA1c levels. This was also consistent with the nature of observational studies.

### Variables

Dependent variables: Montreal Cognitive Assessment, (MoCA) Santa Barbara Sense of Direction Scale (SBSOD), Time and Go (TUG) test, TUG test under dual-task conditions (cognitive-motor and motor-motor), reaction time, Fatigue Assesment Scale (FAS), HbA1c, Serum Ferritin, Thyroid Stimulating Hormone (TSH), B12 and C-Reactive Protein (CRP) levels, and vitamin D. Independent variables: Participants’ T2DM status. Control variables: Age, gender, education level, and participants’ comorbidity presence.

### Data collection process

Data were collected through demographic questionnaires, self-report scales, clinical examinations, and laboratory records. All assessments were performed by three previously trained, experienced physiotherapists, following standardized protocols. The physiotherapists were blinded to participants’ glycemic classification throughout all functional testing procedures. Group allocation was determined independently by endocrinologists based on laboratory findings and was not disclosed to the assessors during data collection. Laboratory tests were analyzed in the hospital’s accredited biochemistry laboratory. In addition, missing data occurring during the data collection process were handled using multiple imputation procedures in SPSS (IBM SPSS Statistics, Version 25.0). The proportion of missing data was less than 5%. Inapplicable tests were coded as “not applicable” and excluded from the relevant analyses.

### Data collection tools

Participants were asked to complete a 15-question descriptive information form. The questions in this form were prepared based on relevant literature [3, 5, 7, 9]. The MoCA, SBSOD, TUG test, TUG test under dual-task conditions (cognitive-motor and motor-motor), Nelson-Foot Reaction Time, FAS, HbA1c, Serum Ferritin, TSH, B12 and CRP levels and vitamin D were assessed.

### MoCA

The MoCA is a short, simple, and practical 30-question scale that assesses seven different cognitive domains, including visuospatial skills, executive functions, memory, attention, language, abstract thinking, and orientation. The MoCA was developed by Nasreddine et al. [15] and its Turkish validity and reliability were conducted by Selekler et al. [16]. The maximum total score on the MoCA is 30. In the Turkish version of the MoCA, a score below 25 indicates cognitive impairment.

### SBSOD

The SBSOD is a 15-question questionnaire with self-assessed responses ranging from 1 (completely agree) to 7 (completely disagree) to measure individuals’ spatial navigation abilities. The SBSOD is scored between a minimum of 15 and a maximum of 105. High scores indicate that individuals have a good sense of direction. The SBSOD was developed by Hegarty et al., and its Turkish adaptation was conducted by Turgut [17].

### TUG

The TUG is a simple physical performance test that assesses individuals’ physical function. Individuals are asked to get up from a standard chair, walk 3 m, turn around, and then return to the chair and sit down. The duration of this entire exercise is recorded in seconds. TUG was developed by Podsiadlo and Richardson [18].

### TUG test under dual-task conditions (cognitive-motor and motor-motor)

Cognitive-motor dual-task performance is the ability to perform both a cognitive and a motor task simultaneously. In this study, participants simultaneously completed the counting days of the week (cognitive task) and the TUG test (motor task). Participants’ times were then recorded with a stopwatch. Motor-motor dual-task performance is the ability to perform two motor tasks simultaneously. In this study, participants were given the task of carrying four half-liter bottles and the TUG test simultaneously. Participants’ performance times were recorded with a stopwatch.

### Nelson-foot reaction time

This test is used to assess the motor response and reflexes of an individual’s lower extremity. A ruler is placed on the participant’s foot, and the ruler is quickly grasped with the toe. The reaction time is recorded by reading from the ruler.

### FAS

This scale is a self-reported assessment developed to assess individuals’ fatigue levels. The FAS consists of 10 questions and has a 5-point Likert-type rating, from 1:never 5:always. The minimum score for the FAS is 10 and the maximum is 50. Participants with high scores indicate severe fatigue. This scale was developed by Michielsen et al. [19] and its Turkish reliability was determined by Ozkeskin et al. [20].

### Bias and control strategies

Preventing bias at every stage, from study planning to data collection and analysis, is crucial for the validity and generalizability of scientific results [21]. Based on this principle, selection bias was reduced by using a sequential sampling method when selecting participants for the study. The assessments were conducted by three different physiotherapists, and the absence of information about the participants’ group affiliation prevented observer bias by blinding the assessor. The implementation of standardized test and assessment protocols used in the study, along with their high validity and reliability, prevented measurement bias. To prevent response bias, patients were volunteered using neutrally worded questions, avoiding directives. Data entry errors were double-checked. Cross-checking was also performed on randomly selected datasets.

### Sample size and power analysis

In the study, a priori power analysis was performed using a fixed-effect one-way ANOVA for four independent groups using the G*Power 3.1.9.4 program (Heinrich Heine University Düsseldorf, Germany). Based on a similar study [5], where the effect size was referenced (f = 0.51), a 5% significance level (α = 0.05), and 95% statistical power (1-β = 0.95), the total sample size required to detect significant group differences was calculated as 72.

### Statistical analysis

The data were analyzed using the SPSS version 25.0 (Statistical Package for the Social Sciences) (IBM Corp., Armonk, NY, USA). The normality of continuous variables was assessed using the Kolmogorov–Smirnov test. Data with a normal distribution were expressed as mean, standard deviation (SD), and minimum-maximum while non-normally distributed data were presented as median and interquartile range (IQR). Number (n) and percentage (%) were used for categorical variables. For normally distributed data, comparisons of quantitative variables across four independent groups were performed using one-way ANOVA. For non-normally distributed data, between-group comparisons were conducted using the Kruskal-Wallis test. The Mann–Whitney U test was applied for pairwise comparisons. The level of significance was set at *p* < 0.05 for comparisons among the four groups. Bonferroni correction was applied for pairwise comparisons, and the level of statistical significance set at *p* < 0.0125.

To further evaluate whether the observed group differences were independent of potential demographic and clinical confounding factors, additional adjusted analyses were conducted using analysis of covariance (ANCOVA). Age, BMI, educational level, and comorbidity status were included as covariates because of their established influence on cognitive and physical performance in older adults. ANCOVA models were performed only for outcomes demonstrating significant between-group differences in the primary analyses. Prior to the adjusted analyses, assumptions of ANCOVA were evaluated. Homogeneity of regression slopes was assessed by examining the interaction terms between group and covariates, and homogeneity of variance was assessed using Levene’s test. No significant assumption violations were observed. Adjusted group effects were reported using F statistics, p values, and partial eta squared (η²p) effect sizes.

## Results

### Participant characteristics

A total of 94 participants, including 53 female (56.4%) and 41 male (43.6%), were included in the analysis. The participants had a mean age of 67.12 ± 5.84 years, a mean height of 1.63 ± 0.08 m, a mean weight of 79.31 ± 14.06 kg, and a mean body mass index (BMI) of 29.73 ± 4.77 kg/m². Based on HbA1c values, participants were divided into four groups: Group 1 = non-diabetic, Group 2 = prediabetes, Group 3 = controlled diabetes, and Group 4 = uncontrolled diabetes. The gender distribution, age, height, weight, BMI, and HbA1c levels of the four groups are presented in Table 1. As expected, there was a difference only in HbA1c levels among the groups (*p* < 0.001).

**Table 1:** Distribution of gender, age, height, weight, BMI, and HbA1c values among groups

	Group 1	Group 2	Group 3	Group 4	
	(Non-Diabetic)	(Prediabetes)	(Controlled Diabetes)	(Uncontrolled Diabetes)	
	*n* = 27	*n* = 23	*n* = 20	*n* = 24	
Gender	**n (%)**	**n (%)**	**n (%)**	**n (%)**	**Test Statistics (p)**
Female	15 (55.60)	13 (56.50)	11 (55.00)	14 (58.30)	0.996^**a**^
Male	12 (44.40)	10 (43.50)	9 (45.00)	10 (41.70)	0.996^**a**^
	**Median (IQR)**	**Median (IQR)**	**Median (IQR)**	**Median (IQR)**	
Age	66 (11)	66 (11)	66 (8)	67.50 (10)	0.372^**b**^
Height (m)	1.65 (0.09)	1.63 (0.12)	1.65 (0.11)	1.59 (0.14)	0.401^**b**^
Weight (kg)	74.20 (21)	75 (25)	83.50 (20)	80 (17)	0.165^**b**^
HbA1c	5.30 (0.50)	6.00 (0.20)	6.70 (0.20)	8.65 (2.25)	**< 0.001** ^**b**,^ *
	**Mean ± SD**	**Mean ± SD**	**Mean ± SD**	**Mean ± SD**	
BMI (kg/m²)	27.78 ± 3.51	29.83 ± 5.64	30.92 ± 5.27	30.83 ± 4.22	0.070^**c**^

^a^Chi-Square Test, ^b^Kruskal-Wallis Test, ^c^One-Way ANOVA, *BMI*=Body mass index, *IQR*=Interquartile Range, *SD*=Standard deviation, *p < 0.001

### Cognitive functioning and spatial orientation

MoCA scores were compared between groups, and significant differences were observed in the attention (*p* = 0.001) and delayed recall (*p* = 0.001) subdomains and the total score (*p* < 0.001). There were no differences between groups in the other MoCA subdomains or in SBSOD scores (*p* > 0.05). In pairwise comparisons, attention subdomain of MoCA indicated significant differences between Group 1 vs. Group 3 (*p* = 0.006), Group 1 vs. Group 4 (*p* < 0.001) and Group 3 vs. Group 4 (*p* = 0.008). The delayed recall subdomain of MoCA showed significant differences between Group 1 vs. Group 4 (*p* < 0.001). The total scores of MoCA indicated significant differences between Group 1 vs. Group 4 (*p* < 0.001) and Group 3 vs. Gorup 4 (*p* = 0.001). Comparison of MoCA and SBSOD scores among groups are presented in Table 2.

**Table 2:** Comparison of MoCA and SBSOD scores among groups

	Group 1			Group 2		Group 3			Group 4			
	(Non-Diabetic)			(Prediabetes)		(Controlled Diabetes)			(Uncontrolled Diabetes)			
Between Group Comparisons	*n* = 27			*n* = 23		*n* = 20			*n* = 24			
**MoCA**	**Median**	**IQR**		**Median**	**IQR**	**Median**		**IQR**	**Median**	**IQR**		**p** ^**a**^
Visuospatial and executive	4	2		4	2	4		2	4	1		0.280
Naming	2	1		2	1	3		1	2	1		0.225
Attention	6	1		4	3	5		1	4	1		0.001*
Language	2	2		2	1	1		1	1	2		0.052
Abstraction	2	0		2	1	2		1	2	1		0.077
Delayed recall	3	2		2	3	2		2	0	2		0.001*
Orientation	6	0		6	0	6		0	6	0		0.342
Total score	25	6		22	7	22		4	18.50	6		< 0.001*
**SBSOD**	81	18		72	46	63.50		33	63	47		0.057
	**MoCA**			**MoCA**		**MoCA**						
	**Attention Subscale**			**Delayed Recall Subscale**		**Total Score**						
**Pairwise Comparisons**	**z**	**p** ^**b**^		**z**	**p** ^**b**^	**z**		**p** ^**b**^				
Group 1 – Group 2	−1.821		0.069	−2.093	0.036		−1.908	0.056				
Group 1 – Group 3	−2.762		0.006**	−1.946	0.052		−1.988	0.047				
Group 1 – Group 4	−3.497		< 0.001**	−3.854	< 0.001**		−3.850	< 0.001**				
Group 2 – Group 3	−0.324		0.746	−0.448	0.654		−0.061	0.951				
Group 2 – Group 4	−1.539		0.124	−1.818	0.069		−2.411	0.016				
Group 3 – Group 4	−2.645		0.008**	−2.360	0.018		−3.176	0.001**				

^a^Kruskal-Wallis Test, ^b^Mann-Whitney U Test, *MoCA*=Montreal Cognitive Assessment, *SBSOD*=Santa Barbara Sense of Direction Scale, *IQR*=Interquartile Range, *p<0.05 ** *p*<0.0125 (Bonferroni correction applied in pairwise comparisons)

### Motor and dual-task performance

TUG, dual-task TUG, and foot reaction time scores were compared among groups (Table 3). Significant differences were found in the TUG (*p* = 0.003), TUG test under dual-task (motor-motor) (*p* = 0.022), TUG test under dual-task (cognitive-motor) (*p* = 0.029), left foot reaction time (*p* = 0.002) and right foot reaction time (*p* < 0.001). In pairwise comparisons, the TUG test indicated significant differences between Group 1 vs. Group 3 (*p* = 0.003) and Group 1 vs. Group 4 (*p* = 0.004). No significant differences were observed in pairwise comparisons for dual-task TUG performance (*p* > 0.0125). The foot reaction time-left revealed significant differences between Group 1 vs. Group 2 (*p* = 0.010) and Group 1 vs. Group 4 (*p* = 0.001). The foot reaction time-right revealed significant differences between Group 1 vs. Group 2 (*p* = 0.002), Group 1 vs. Group 3 (*p* = 0.003) and Group 1 vs. Group 4 (*p* < 0.001).

**Table 3:** Comparison of TUG, Dual-task TUG, and Foot Reaction Time among groups

	Group 1		Group 2		Group 3		Group 4		
	(Non-Diabetic)		(Prediabetes)		(Controlled Diabetes)		(Uncontrolled Diabetes)		
	*n* = 27		*n* = 23		*n* = 20		*n* = 24		
**Between Group Comparisons**	**Median**	**IQR**	**Median**	**IQR**	**Median**	**IQR**	**Median**	**IQR**	**p** ^**a**^
TUG	6.22	2.61	7.15	1.92	8.37	1.98	8.46	3.65	0.003*
TUG + Motor Task	6.68	2.97	7.37	2.20	8.19	2.49	8.68	1.95	0.022*
TUG + Cognitive Task	8.92	5.15	9.52	4.02	11.09	5.14	11.21	6.20	0.029*
Foot Reaction Time - Left	21.20	7.70	25.70	10.01	24.65	7.50	28.50	9.90	0.002*
Foot Reaction Time - Right	19.00	7.30	25.60	10.00	24.85	7.60	27.75	11.30	< 0.001*
	**TUG**			**TUG + Motor Task**			**TUG + Cognitive Task**		
**Pairwise Comparisons**	**z**	**p** ^**b**^		**z**		**p** ^**b**^	**z**	**p** ^**b**^	
Group 1 – Group 2	−1.441	0.150		−0.457		0.647	−0.847	0.397	
Group 1 – Group 3	−2.948	0.003**		−2.324		0.020	−2.431	0.015	
Group 1 – Group 4	−2.869	0.004**		−2.010		0.044	−2.350	0.019	
Group 2 – Group 3	−1.960	0.050		−2.045		0.041	−1.802	0.072	
Group 2 – Group 4	−1.969	0.049		−2.064		0.039	−1.852	0.064	
Group 3 – Group 4	−0.283	0.777		−0.247		0.805	−0.283	0.777	
	**Foot Reaction Time - Left**					**Foot Reaction Time - Right**			
**Pairwise Comparisons**	**z**			**p** ^**b**^		**z**		**p** ^**b**^	
Group 1 – Group 2	−2.571			0.010**		−3.175		0.002**	
Group 1 – Group 3	−1.787			0.074		−2.928		0.003**	
Group 1 – Group 4	−3.455			0.001**		−3.681		< 0.001**	
Group 2 – Group 3	−0.804			0.421		−0.573		0.567	
Group 2 – Group 4	−1.288			0.198		−0.447		0.655	
Group 3 – Group 4	−1.887			0.059		−0.908		0.364	

^a^Kruskal-Wallis Test, ^b^Mann-Whitney U Test, *TUG*=Time Up and Go Test, *IQR*=Interquartile Range, *p < 0.05 ** *p* < 0.0125 (Bonferroni correction applied in pairwise comparisons)

### Fatigue and biochemical markers

FAS and biochemical markers were compared among groups (Table 4). Significant differences were found in the physical (*p* < 0.001), mental (*p* < 0.001) subscales and total scores of FAS (*p* < 0.001). Among the biochemical markers, only fasting blood glucose levels showed a statistically significant difference among the groups (*p* < 0.001). In pairwise comparisons, the physical, mental subscales and total scores of FAS and fasting blood glucose revealed significant differences between Group 1 vs. Group 2, Group 3 and Group 4 (*p* < 0.0125). No significant differences were observed in pairwise comparisons among the diabetic groups (Groups 1, 2, and 3) (*p* > 0.0125).

**Table 4:** Comparison of FAS and biochemical markers among groups

	Group 1		Group 2		Group 3			Group 4			
	(Non-Diabetic)		(Prediabetes)		(Controlled Diabetes)			(Uncontrolled Diabetes)			
Between Group Comparisons	*n* = 27		*n* = 23		*n* = 20			*n* = 24			
**FAS**	**Median**	**IQR**	**Median**	**IQR**	**Median**	**IQR**		**Median**		**IQR**	
Physical	9	6	16	7	14.5	6		15		6	
Mental	7	3	11	6	11.5	7		13		7	
Total	15	8	25	13	26	12		28.5		12	
**Fasting Blood Glucose**	92	20	113	48	118	38.5		138		105.75	
**Vitamin B12**	322	230	306	169	352	301		409		680	
**Ferritin**	34.30	40.20	42.30	37.90	30.95	38.83		35.30		39	
**Thyroid Stimulating Hormone**	1.67	1.43	0.90	1.94	1.33	2.01		1.41		1.59	
**C-reactive Protein**	2.80	2	4.13	5.80	3.25	5.68		3.30		3.97	
**25-Hydroxy Vitamin D**	14	13	19	16.09	17.50	17.50		13.95		14.14	
**Between Group Comparisons**	**Mean ± SD**	**Min-Max**	**Mean ± SD**	**Min-Max**	**Mean ± SD**	**Min-Max**		**Mean ± SD**		**Min-Max**	
Hemoglobin	13.66 ± 0.28	9.70–16.90	14.03 ± 0.32	11.00–17.80	13.70 ± 0.34	11.50–17.90		14.17 ± 0.28		11.80–17.30	
	**FAS**		**FAS**		**FAS**			**Fasting Blood Glucose**			
	**Physical Subscale**		**Mental Subscale**		**Total Score**						
Pairwise Comparisons	**z**	**p** ^**c**^	**z**	**p** ^**c**^	**z**	**p** ^**c**^		**z**		**p** ^**c**^	
Group 1 – Group 2	−3.830	< 0.001**	−3.493	< 0.001**	−3.647		< 0.001**	−3.067		0.002**	
Group 1 – Group 3	−3.921	< 0.001**	−3.576	< 0.001**	−4.040		< 0.001**	−3.606		< 0.001**	
Group 1 – Group 4	−4.547	< 0.001**	−4.426	< 0.001**	−4.647		< 0.001**	−4.937		< 0.001**	
Group 2 – Group 3	−0.440	0.660	−0.491	0.623	−0.024		0.981	−0.353		0.724	
Group 2 – Group 4	−0.566	0.572	−1.548	0.122	−1.045		0.296	−2.321		0.020	
Group 3 – Group 4	−0.946	0.344	−0.946	0.344	−1.039		0.299	−1.933		0.053	

^a^Kruskal-Wallis Test, ^b^One-Way ANOVA Test, ^c^Mann-Whitney U Test, *FAS*=Fatigue Assessment Scale, *IQR*=Interquartile Range, *SD*=Standard deviation, *Min*=Minimum, *Max*=Maksimum, *p<0.05 **p<0.0125 (Bonferroni correction applied in pairwise comparisons)

### Adjusted group comparisons for primary outcomes

Additional adjusted ANCOVA analyses demonstrated that group differences in cognitive performance, reaction time, and fatigue outcomes remained significant after adjustment for age, BMI, educational level, and comorbidity presence, whereas TUG-related mobility differences were attenuated (Table 5).

**Table 5:** Adjusted ANCOVA analyses for primary outcomes

Outcome	F (group effect)	*p*	Partial η²
MoCA total	8.95	< 0.001	0.228
MoCA Attention	5.17	0.002	0.146
MoCA Delayed recall	5.09	0.003	0.144
Left foot reaction time	7.34	< 0.001	0.195
Right foot reaction time	5.35	0.002	0.150
FAS total	7.42	< 0.001	0.197
FAS physical	6.83	< 0.001	0.184
FAS mental	5.71	0.001	0.158
TUG	1.96	0.126	0.061
TUG + Motor Task	2.11	0.104	0.066
TUG + Cognitive Task	2.13	0.102	0.066

Analysis of Covariance (ANCOVA), *MoCA*=Montreal Cognitive Assessment, *FAS*=Fatigue Assessment Scale, *TUG*=Timed Up and Go Test, *η²p*=Partial Eta Squared. ANCOVA models were adjusted for age, *BMI* educational level, and comorbidity presence. Only outcomes demonstrating significant between-group differences in the primary analyses were included in the adjusted models

## Discussion

The present study demonstrated that uncontrolled T2DM has a critical impact on cognitive functions in older individuals, particularly in the uncontrolled phase. No significant differences were observed in spatial ability, one of the sub-dimensions of the MoCA scale. However, impairments were detected in the attention, delayed recall sub-dimensions, and total MoCA performance. In pairwise comparisons, uncontrolled T2DM was found to consistently show worse cognitive performance compared to both healthy and controlled T2DM patient groups. These findings are consistent with the literature showing that persistent hyperglycemia, insulin resistance, and microvascular and neuroinflammatory changes associated with T2DM affect executive and memory networks in older adults [22, 23]. In contrast, no significant differences were observed in the visuospatial and executive function, naming, language, abstraction, and orientation MoCA subdomains or in spatial ability as assessed via SBSOD. This suggests that some cognitive domains may be less affected by changes associated with early or moderate diabetes [24]. Furthermore, the fact that SBSOD is a self-reported assessment may have limited the detection of subtle spatial impairments. Overall, these findings suggest that older adults with uncontrolled T2DM may have a greater impact on areas of attention and memory.

The study demonstrated that older adults with T2DM, especially those with uncontrolled diabetes, revealed significant impairments in functional mobility and motor performance. Although significant overall group differences were observed in dual-task TUG performance, these differences did not remain significant in pairwise comparisons after Bonferroni correction. Furthermore, in the adjusted ANCOVA analyses, TUG-related mobility outcomes were attenuated after controlling for age, BMI, educational level, and comorbidity presence. Therefore, the dual-task findings should be interpreted cautiously. Rather than indicating definitive between-group impairments, these results may reflect subtle trends toward reduced cognitive-motor performance across the dysglycemia spectrum. It is also possible that the relatively modest subgroup sample sizes and the conservative nature of Bonferroni correction reduced the ability to detect significant pairwise differences. Furthermore, prolonged reaction times in both feet were observed even in the prediabetes group, indicating early motor slowing prior to overt T2DM diagnosis. These results are consistent with recent literature showing that diabetes-related complications, including peripheral neuropathy, vascular changes, and muscle weakness, contribute to reduced mobility and delayed motor responses in older adults [25–27]. The presence of minor deficits in prediabetic individuals aligns with evidence that early metabolic dysregulation can affect neuromuscular performance before significant hyperglycemia develops [28]. Overall, these findings emphasize the importance of monitoring mobility and motor function across the spectrum of glycemic control. Early motor slowing and delayed reaction times, even in prediabetic individuals, suggest that functional decline may begin before overt diabetes is diagnosed, suggesting a potential window for early clinical monitoring and future research. Impaired mobility and slower motor responses can increase the risk of falls, limit participation in daily activities, and reduce overall autonomy, which are key determinants of health-related quality of life in aging populations [4, 11]. Assessment of functional mobility, reaction time, and dual-task performance may help identify subtle deficits that might otherwise go unnoticed and may represent promising targets future longitudinal and interventional studies. Further studies are needed to determine whether incorporating these measures into routine clinical practice provides additional benefit for older adults across the dysglycemia spectrum.

Fatigue emerged as a common problem in both prediabetes and diabetes groups, with higher scores on physical and mental fatigue subscales compared to healthy controls. This pattern suggests that fatigue in older adults with dysglycemia may affect not only physical but also cognitive domains [29]. The observed increase in physical and mental fatigue levels from non-diabetic participants to participants with uncontrolled diabetes supports the impact of impaired glucose regulation on overall functional capacity. These findings are consistent with studies reporting the effects of dysglycemia on mental fatigue, cognitive performance, physical performance, and cognitive endurance, and demonstrate that fatigue is a clinically significant consequence of dysglycemia in older adults [30–32].

In the present study, among the biochemical parameters evaluated, only fasting blood glucose levels showed significant differences. However, this finding was expected to some extent, as participant classification was based on HbA1c-defined glycemic status. In contrast, serum ferritin, TSH, vitamin B12, CRP, and vitamin D did not reveal significant difference across groups. These findings should be interpreted cautiously, as such biomarkers are influenced by multiple age-related, metabolic, inflammatory, nutritional, and lifestyle-related factors beyond glycemic status alone. This finding may be explained by the fact that these biomarkers are affected by numerous factors, including glycemic status as well as age-related changes, inflammation, nutritional status, and lifestyle [23, 33–37]. Therefore, factors other than glycemic status should also be considered when interpreting biochemical findings.

The present study has some limitations. First, the cross-sectional design of the study prevents the determination of causal relationships between glycemic status and observed functional impairments. Longitudinal studies would be necessary to establish temporal links and progression patterns of cognitive and motor decline in prediabetes and T2DM. Second, the sample size in each subgroup, particularly the uncontrolled T2DM group, was relatively modest, which may limit statistical power and the generalizability of the findings to broader populations. Third, the reliance on subjective measures, such as the SBSOD for spatial ability and self-reported fatigue questionnaires, may introduce reporting bias and limit sensitivity in detecting subtle impairments. Fourth, the dual-task assessments, although valuable for exploring cognitive-motor interactions, did not remain statistically significant after correction for multiple comparisons, suggesting that either the task complexity or sample size may have constrained the ability to detect true differences. Fifth, the study cohort consisted exclusively of community-dwelling older adults; thus, results may not be generalizable to institutionalized individuals, those with more severe diabetes complications, or populations from different cultural or ethnic backgrounds. Finally, certain biochemical markers such as ferritin, vitamin B12, TSH, CRP, and vitamin D are influenced by multifactorial determinants, including age-related physiological changes, comorbidities, and lifestyle factors, which may have masked potential differences attributable to glycemic status. Finally, certain potential important clinical variables were not systematically assessed within the scope of this study. Factors such as duration of diabetes, antidiabetic treatment type, physical activity level, diabetic neuropathy status, and polypharmacy may substantially influence study results. Therefore, these variables could not be incorporated into the adjusted analyses, and residual confounding cannot be completely excluded. These limitations should be considered when interpreting the findings and underscore the need for cautious extrapolation to other populations.

This study offers a multidimensional evaluation of older adults across the glycemic spectrum, integrating cognitive, motor, fatigue, and biochemical assessments, which provides a holistic understanding of the functional impact of dysglycemia. The combination of objective performance-based measures (e.g., TUG, dual-task tests, reaction time) with validated cognitive and fatigue scales enhances both the sensitivity and clinical relevance of the findings. By including prediabetic and uncontrolled T2DM participants, the study highlights early and progressive impairments, offering insights into potential areas for future longitudinal research aimed at tracking functional decline and evaluating intervention efficacy.

## Conclusion

In conclusion, uncontrolled T2DM in older adults is associated with impairments in cognitive function, particularly in attention and memory, as well as reduced functional mobility and slower motor performance. Fatigue, both physical and mental, is elevated across prediabetic and individuals with T2DM, reflecting the multidimensional impact of dysglycemia on daily functioning. Among the evaluated biochemical parameters, only fasting blood glucose differed between groups, likely reflecting differences in glycemic status. Future longitudinal studies with larger and more diverse cohorts may be beneficial to clarify causal relationships, track the progression of functional and cognitive decline, and evaluate the effectiveness of targeted interventions aimed at preserving mobility, cognitive function, and quality of life in older adults with dysglycemia.

## Data Availability

The data that support the findings of this study are available from the corresponding author upon reasonable request.
